# wFleaBase: the *Daphnia *genome database

**DOI:** 10.1186/1471-2105-6-45

**Published:** 2005-03-07

**Authors:** John K Colbourne, Vasanth R Singan, Don G Gilbert

**Affiliations:** 1 Center for Genomics and Bioinformatics, Indiana University, Bloomington, Indiana USA; 2 School of Informatics, Indiana University, Bloomington, Indiana USA; 3 Department of Biology, Indiana University, Bloomington, Indiana USA

## Abstract

**Background:**

wFleaBase is a database with the necessary infrastructure to curate, archive and share genetic, molecular and functional genomic data and protocols for an emerging model organism, the microcrustacean *Daphnia*. Commonly known as the water-flea, *Daphnia*'s ecological merit is unequaled among metazoans, largely because of its sentinel role within freshwater ecosystems and over 200 years of biological investigations. By consequence, the *Daphnia *Genomics Consortium (DGC) has launched an interdisciplinary research program to create the resources needed to study genes that affect ecological and evolutionary success in natural environments.

**Discussion:**

These tools include the genome database wFleaBase, which currently contains functions to search and extract information from expressed sequenced tags, genome survey sequences and full genome sequencing projects. This new database is built primarily from core components of the Generic Model Organism Database project, and related bioinformatics tools.

**Summary:**

Over the coming year, preliminary genetic maps and the nearly complete genomic sequence of *Daphnia pulex *will be integrated into wFleaBase, including gene predictions and ortholog assignments based on sequence similarities with eukaryote genes of known function. wFleaBase aims to serve a large ecological and evolutionary research community. Our challenge is to rapidly expand its content and to ultimately integrate genetic and functional genomic information with population-level responses to environmental challenges. URL: .

## Background

The micro-crustacean *Daphnia *is a ubiquitous resident of inland waters within all continents of the globe and is the subject of study for numerous biological disciplines including limnology, ecology, physiology, toxicology, population genetics and evolution. Many attributes make this organism an ideal model for ecological and evolutionary genomics research. As the principal grazers of algae and the primary forage of fish, *Daphnia *are key members of aquatic food webs and are easily sampled in great numbers. These animals inhabit remarkably diverse environments and show striking patterns of convergent evolution linked to specific habitat transitions [1]. Their mode of reproduction (cyclical parthenogenesis) is convenient for experimental genetics, providing both long-term clonal lineages and controlled outbred populations by manipulating the environmental cues required for the induction of male production and for mating [2]. Yet most notably, *Daphnia *offer unprecedented opportunities to study historical responses to environmental change, by harvesting, dating and resurrecting annually sedimented diapausing eggs within lacustrine basins and by competing past products of evolution against their modern descendents [3,4]. For these reasons, an international network of investigators is creating community resources, already proven to effectively promote genomic-scale investigations in other disciplines (molecular, cell and developmental biology), with a goal to understand connections between genome structure, gene expression, individual fitness and population-level responses to environmental challenges.

wFleaBase is a project of the *Daphnia *Genomics Consortium [5] and is designed to be a resource where users can search and retrieve sequence data for genes of ecological importance, or find putative genes modulating traits of interest based on their homologies to functionally characterized genes in other model organisms. Therefore, wFleaBase is an organized repository of *Daphnia *specific sequences with standard bioinformatic tools to facilitate gene discovery. This function includes BLAST analyses and links to gene reports for other eukaryotic genomic models via euGenes [6]. However, for most of these other model species, characterized genes are ineluctably biased toward those sets whose phenotypic effects are observed in the benign settings of a laboratory. With the additional goal of elucidating the function of novel genes with environment-specific expression patterns, wFleaBase is also designed to help locate genes with no known functions. For this purpose, a pipeline of bioinformatic tools is created to supply DNA markers from raw sequence trace files. Genetic map information on the location of variable DNA markers will soon be presented, allowing researchers to systematically screen genomic regions for the presence of quantitative trait loci (QTL) by using the available markers in their studies. Finally, to facilitate subsequent gene-specific capture by positional cloning, catalogues of available arrayed DNA libraries are displayed within the DGC web pages.

The main sources of data for wFleaBase are direct submissions from DGC members and from research at large genome sequencing centers. The latest data can be accessed by web browser at  and Internet file transfer at .

## Construction and content

### Generic genome database

This service is built using tested genome database components and open source software that are shared in common with several other databases. Middleware in Perl and Java are added to bring together BLAST, sequence reports, searches and other bioinformatics programs for web access. The Indiana University Genome Informatics Laboratory houses wFleaBase, along with related genome databases FlyBase [7] and euGenes [8]. In the last two years, this work is coalescing with sister organism database projects under the umbrella of Generic Model Organism Database project (GMOD [9]). The relational database from GMOD [10] used for FlyBase and wFleaBase is named Chado (after "the Way of Tea" ceremony). It includes a schema for structuring a growing range of genome information, works with the free PostgreSQL database package (among others), and includes a Chado XML exchange format and tools. Significantly, a community of bioinformaticians is sharing development and use of these components. Another project that has made wFleaBase simple to start is Argos (D.G. Gilbert et al., in preparation [11]), a framework for building and distributing genome databases, with pre-configured core components listed in Table 1. A third basic GMOD component of wFleaBase is LuceGene (D.G. Gilbert et al., in preparation), which provides rapid, data object-oriented searches, with data and document retrieval of a wide range of genome information.

To start wFleaBase, we copied a genome database/web server template from Argos infrastructure, including Chado database and genome informatics tools, loaded the database with a first set of sequences, and produced BLAST comparisons of these against 10 other eukaryote genomes from the euGenes project. The euGenes project provides a standard summary of gene and genome information from eukaryotic organisms, and includes over 200,000 named genes and their functions, with 900,000 genome features. This eukaryote genome collection allows new genomes (like *Daphnia*'s) to be matched by sequence similarity, then annotated with reference gene information. When *Daphnia *or other new organism sequences are matched to this data, it suggests their gene function and provides starting points for experiments into their ecological and evolutionary genetic significance.

### wFleaBase records and accession numbers

Sequences are assigned unique and stable accession numbers upon entry into the Chado database, which are organized into seven divisions according to whether they are derived from genome survey sequences (GSS), expressed sequence tags (EST) or high-throughput genomic (HG) and cDNA (HC) projects. *Daphnia *sequences from other public databases (PB), mitochondrial sequences from molecular systematic studies of the genus (MT) and amplicons of microsatellite DNA markers (MS) are also categorized. To date, wFleaBase contains 14,451 records, including EST (WFes0000001-WFes0012408), GSS (WFgs0000001-WFgs0001495) and MS (WFms0000001-WFms0000548) sequences. Each sequence header provides a short description on the type of sequence, the species and strain from which the sequence was obtained, the library identification code for the cloned fragment with synonyms and contact name. Full contact information is provided elsewhere [12]. For convenience, sequences can be downloaded in fasta format from each division via the FTP service or by navigating to specific Data sub-directories of the Genomics hyperlink, which is printed on the side menu of the web pages. At this moment, researchers are requested to send new *Daphnia *sequences to the corresponding author, for processing and archiving the data into wFleaBase. Submissions undergo quality assurance checks for vector contamination and correct taxonomy before they enter the database.

## Utility

### Gene searching and discovery

As an information and gene discovery system, wFleaBase focuses on providing efficient tools for searching and retrieving records of interest. Its current features are best highlighted by co-navigating the web pages along with a user interested in locating ecologically relevant genes, for example, genes that confer resistance to elevated levels of ultra-violet radiation encountered by closely related species to *D. pulex*. Beginning at the welcome page, the user can navigate via the hyperlink located at the top menu towards the Blast page of wFleaBase  to perform sequence-similarity searches on the archived data using the BLAST family of programs. The user enters a nucleotide sequence, whose gene function is well characterized and evolutionarily conserved, with a goal to find the homologous gene in *Daphnia*. For example, a *Drosophila melanogaster *mRNA sequence obtained from GenBank (NM 165564) or from FlyBase (FBgn0003082) for the gene *photorepair *is used to query all *Daphnia *sequences using the default settings of the tblastx program. Alternatively, the user can select to query species-specific GSS or EST databases. This search retrieves record WFgs0000440, which is a 917 nucleotide sequence with a best match score of 83 bits and an E-value of 5e-45. Using this information, the user can then download the *Daphnia *sequence onto their personal computers as a text file, design primers using their own software to probe the arrayed *Daphnia *cosmid library by the Polymerase Chain Reaction (PCR), identify bacterial clones containing the gene, and characterize the entire locus by sequencing. Indeed, this specific exercise identifies at least three cosmids (out of 37,000) containing a likely homologue to *photorepair *from *Drosophila *[13].

Returning to the welcome page, the user can instead choose to explore tables containing data extracted from automated BLAST searches against the euGenes database, which includes annotated genome sequences from 10 eukaryotic model organisms. Although this option for gene searching is more tedious, it does allow users to focus precisely on the data currently available in wFleaBase. Four tables of BLAST results are offered at  by following the "Genomics" hyperlinks located at the top and side menus. At present, *Daphnia *EST and GSS sequences are each compared to the protein coding genes and to genomic sequences in euGenes. Many options exist for sorting the BLAST tables. The user can specify what BLAST result columns to show, and can sort these columns based on the ascending or descending order of their entries. The tables can also include BLAST results against all organisms within euGenes or the tables can be filtered to include results from comparisons against a single taxon. For example, the same user, now looking to find a *Daphnia *homologue to genes known to confer salt-resistance to species inhabiting saline environments, begins by searching for names or euGenes accession numbers of functionally related genes within the Blast tables using the wFleaBase search function located in the top menu of the Blast tables. If the user chooses to search for "ATPα", which is a sodium/potassium-exchanging ATPase shown to be under positive selection in brine shrimp populations adapted to ultra-saline waters [14], 11 EST records that match ATPα in fly are discovered with bit scores and E-values ranging from 42.36 and 0.002 to 327.0 and 2.2e-89. The user can retrieve the *Daphnia *sequences via hyperlinks located in the first column of the search results, or further uncover the extent of evolutionary conservation for this gene by examining the euGene Reports, also via hyperlinks located in the last column. Alternatively, if the user chooses to use the FlyBase accession number for this gene (FBgn0002921) to retrieve *Daphnia *homologues using the search function, the same 11 records are obtained.

### Tools for hunting unknown genes

Although effective, the candidate gene approach to finding *Daphnia *genes of ecological interest is limited by the levels of sequence and functional conservation among characterized genes in other model organisms. Work is underway by the DGC to create the required tools for identifying ecologically relevant genes by positional mapping using microsatellite markers. wFleaBase presently archives 528 microsatellite markers [15]. Yet, to generate additional loci for genetic mapping in *D. pulex *and *D. magna*, wFleaBase integrates a suite of computational programs that (i) identifies microsatellites from raw DNA sequencer trace files, (ii) designs optimal primers for amplifying the markers and (iii) indexes the amplicon, microsatellite motifs and primer information into the Microsat database [16]. The Microsat database will rapidly grow by applying this pipeline to trace files emerging from the *Daphnia *genome sequencing project.

### The wFleaBase search function

wFleaBase uses LuceGene to support rapid search and retrieval of the sequence database, of Blast table entries, of *Daphnia *Medline references and of *Daphnia *web documents. LuceGene [17], based on the Lucene [18] search system, is an open-source part of the GMOD project. A major benefit of LuceGene is the large variety of data formats that can be added to the search system with minimal work. For instance, currently supported formats used in wFleaBase include Simple text, XML (Medline abstracts and Gene sequence annotation), HTML, Tabular data, Bio-formats (Fasta, GenBank, EMBL) and Gene object data used by euGenes. Search terms such as "magna" to retrieve all sequences from this species, can be entered in a Search box at the head of all web pages. The search is refined at the main wFleaBase search page by specifying the search library (sequences, references, documents or Blast tables) and the library fields containing the queried term. Options are also available to detail the output format, and each result is hyperlinked to the source document for easy access to the data. On a separate web page (Batch download), users can recover multiple records obtained from complex queries and save the results to a file.

## Discussion and conclusions

The wFleaBase project is young. Its future success is linked to the DGC and its goal to develop *Daphnia *into a functional genomics research model, with the added advantage of interpreting observations in the context of natural ecological challenges. wFleaBase is currently designed to facilitate gene discovery for immediate use in research projects. However, the functionality of this service will grow in equal pace with the rapid accumulation of genomics data. Within the next year, this site will host the full genome sequence for *D. pulex *– a collaborative project involving the U.S. Department of Energy Joint Genome Institute, the Environmental Protection Agency and the DGC. Genetic maps for *D. pulex *and *D. magna *are also under construction. Therefore, wFleaBase will soon be enhanced by implementing the CMap module of GMOD and allow users to compare the *Daphnia *genetic and physical maps emerging from current research and to choose the most appropriate set of markers for quantitative trait locus (QTL) mapping projects. Simultaneously, wFleaBase will also assemble cDNA sequences from another large DGC sequencing effort aimed to document most of the *Daphnia *transcriptome. *Daphnia *gene reports will be created in accordance to standards set by current model organism databases [19]. Gbrowse genome browser and Apollo annotation editor will be used for viewing genome features when they are available. Informatics efforts focus on implementing existing database tools rather than development of new ones, providing a cost-effective genome database for these species. In this way, the current format linking *Daphnia *genomic information to other model species will be reinforced, allowing greater opportunities to apply the candidate gene approach for identifying genes of ecological importance.

## Availability and requirements

wFleaBase is publicly available and can be accessed at  using web browsers and at  by using internet file transfer protocols.

## Authors' contributions

JC contributed data, web documentation and aided in the overall design and functionality of this database. VS contributed programming for both the database and Blast searches, and contributed to its design and development. DG contributed the generic database framework design, the overall web structure, the euGenes data and built GMOD database and Blast tools. All authors read and approved the final manuscript.

## Figures and Tables

**Table 1:** GMOD and Argos components used in wFleaBase

*Section*	*Components*
wFleaBase	Data, database files, documents, web tools specific to *Daphnia*
Java	Chado database tools, genome sequence reports, LuceGene search, Ant build system, database interfaces, XML tools, Tomcat web server, Axis web services
Perl	BioPerl, GBrowse, Chado database tools, Cmap comparative maps, database interfaces, Web tools, XML tools
Servers	BLAST (NCBI), Apache web server, PostgreSQL, and BerkeleyDB databases
Systems	Compiled portions for supported operating systems
Install & Root	Common configurations, web server, installation scripts and instructions
